# Unplanned movement tasks requiring rapid decision-making: a scoping review of study characteristics and methodological approaches

**DOI:** 10.1186/s13102-026-01682-0

**Published:** 2026-05-08

**Authors:** Maximilian Siener, Alexandra Unger, Jan Wilke

**Affiliations:** 1 Department of Neuromotorics and Movement, BaySpo – Center of Sport Science, University of Bayreuth, Bayreuth, Germany; 2 Department of Movement Science, Institute of Sports Science, University of Klagenfurt, Klagenfurt, Austria; 3 University of Teacher Education Carinthia, Klagenfurt, Austria

**Keywords:** Biomechanics, Humans, Unanticipated, Kinetic, Kinematic, COD, Cut

## Abstract

**Background:**

The integration of neurocognitive challenges into biomechanical movement tasks has gained attention due to its potential relevance for assessments of physical function, injury risk, and performance. However, a comprehensive mapping of the chosen methodological approaches, targeted populations, and applied outcome measures is still lacking. This scoping review hence aimed to synthesize the current literature on unplanned movement tasks combining cognitive decision-making and biomechanical outcome measurements.

**Methods:**

A systematic literature search following the PRISMA-ScR (Preferred Reporting Items for Systematic Reviews and Meta-Analyses—extension for scoping reviews) guidelines was performed in Web of Science (Core Collection), MEDLINE (PubMed), Cochrane Library, and Google Scholar. Included studies combined unplanned movement tasks (e.g., change-of-direction or stopping) with biomechanical assessments. Eligible articles were analysed in terms of participant characteristics, movement type, unplanned task type, reactive stimulus, and biomechanical outcome variables.

**Results:**

From the total of 167 studies, the majority focused on change-of-direction tasks (82%), mostly using standardized angles of 45° and moderate approach speeds (3.9 ± 0.9 m/s). Jump (7%), land (12%), and/or stop tasks (3%) were less frequent. Most studies (83%) relied on simple visual cues (e.g., lights or symbols), whereas more ecologically valid stimuli (e.g., videos or real opponents) were rarely applied. Biomechanical analyses predominantly focused on knee angles and moments as well as ground reaction forces, while only 23% of studies included electromyography measurements. Older adults (50+ years) were not represented.

**Conclusion:**

Although research on unplanned biomechanical tasks is growing, significant methodological heterogeneity and limited ecological validity may constrain the interpretability and applicability of findings. Future research should aim for task designs that better reflect real-world conditions and include diverse populations and comprehensive neuromuscular assessments.

**Supplementary Information:**

The online version contains supplementary material available at 10.1186/s13102-026-01682-0.

## Introduction

Biomechanical measurements have become commonplace in sports and sports medicine, aiming to gauge the association of kinetic and kinematic variables with a variety of factors such as performance [1], fatigue [2, 3], or and the development or prevention of musculoskeletal injuries [4]. In particular, biomechanical analyses are increasingly used in injury risk screening, rehabilitation monitoring, and return-to-sport decision-making in athletic and clinical populations. Many of the available studies focused on the loads (i.e., joint moments, ground reaction forces) placed on the locomotor system [5] or the forces produced by skeletal muscle during sports-related movement (e.g., running or jumping; [6]). Although the study of movement biomechanics yields highly valuable data, the traditional testing paradigms may be limited in terms of ecological validity.

On the field, athletes engage in a highly complex and rapidly changing environment, continuously adapting their motor plans depending on the actions of teammates and opponents. This implies that cognitive processing, i.e., sustained attention, visual processing, and flexible decision-making, is key to both game-related success and safe sports execution. Importantly, many non-contact sports injuries occur in such unplanned situations, for example during sudden changes of direction or unexpected perturbations. However, a large share of the previous biomechanical research has been conducted under stable and predictable conditions not including reactive components. Examples include jump tests with a pre-specified landing leg or changes of direction (COD) with pre-specified movement directions.

To mimic the unforeseen cognitive challenges during sports practice, more and more studies have recently been performed using unplanned as opposed to pre-planned movement tasks [7, 8]. Here, participants are asked to perform a baseline movement (e.g., fast running) and to spontaneously respond to a visual stimulus (e.g., light/arrow), altering the original motor action (e.g., COD into briefly indicated direction). As participants usually only have a few hundred milliseconds to react, these time-constrained motor-cognitive tasks have been suggested to better reflect typical situations in sport or everyday life [9]. Assessing biomechanical variables such as joint angles or ground reaction forces during unplanned movement may hence be more indicative of risk factors for joints or muscles than pre-planned movement tests [10]. From a clinical and applied perspective, unplanned movement tasks may offer added value for injury risk assessment [11, 12], functional diagnostics [13, 14], and rehabilitation monitoring [15, 16] as well as return-to-sport decision making, as they challenge neuromuscular control under time-constrained and cognitively demanding conditions.

The clinical relevance of unplanned movement tasks has been highlighted in several review articles and meta-analyses [17–22]. For example, Brown et al. [7] and Giesche et al. [18, 19] demonstrated that unplanned movement tasks such as jump landings and cuts, compared to pre-planned tasks, are characterized by greater knee abduction angles, increased tibial internal rotation, and greater ankle plantar- and dorsiflexion. These biomechanical patterns are commonly discussed as being associated with an increased risk of injury [23], e.g., ruptures of the anterior cruciate ligament or ankle sprain. Ebner et al. [20] focused on COD tasks only. These authors found ambiguous evidence for knee valgus angles but higher knee flexion in unplanned vs. planned movements. Importantly, the existing reviews focused on the actual effects of unplanned movements on angles and moments, with a focus on specific joints [18, 19] or movement tasks [7, 20]. Yet, beyond the studies identified in the existing reviews, a substantial number of additional studies—predominantly cross-sectional in design—have investigated unplanned movements across different joints, movement tasks, populations, and measurement approaches. However, to date, no comprehensive overview has systematically mapped this body of literature looking at study methodology. Consequently, it remains unclear which approaches have become established, which populations are represented or underrepresented, which outcomes are addressed insufficiently, and which joints or movement tasks have not yet been examined in sufficient depth.

Against this background, the aim of this scoping review was to comprehensively map the methodological landscape of research on unplanned movement tasks in sports and clinical biomechanics. Specifically, we sought to summarise characteristics of investigated populations, reactive movement paradigms, and biomechanical outcomes, and to identify methodological trends and research gaps. By providing an overview of existing approaches and blind spots, the findings of this review may inform future research and support clinicians and practitioners in selecting and designing reactive movement tasks for injury risk assessment, rehabilitation monitoring, and clinical decision-making.

## Methods

### Protocol and registration

A scoping review was conducted and the protocol was drafted in accordance with the methodological recommendations of Peters et al. [24], the PRISMA Extension for Scoping Reviews [25], and the consensus statement of Ardern et al. [26]. Ethical standards for systematic reviews were followed as outlined by Wager and Wiffen [27]. The review was registered with the International Prospective Register of Systematic Reviews (PROSPERO) on 20 October 2025 (CRD420251171863).

### Sources and search strategy

Two independent investigators (MS and AU) performed a systematic literature search. The databases Web of Science (Core Collection), MEDLINE (via PubMed), the Cochrane Library, and Google Scholar were searched for relevant articles until February 2026. The following search string was used:

- (unplanned OR unanticipated) AND (“change of direction” OR cut* OR land* OR jump* OR stop* OR step*) AND (EMG OR electromyography OR “muscle activity” OR kinetics OR kinematics)

For Google Scholar [28], the search was limited to the first 100 results sorted by relevance [21]. In addition, the reference lists of the articles found were screened for further relevant studies.

### Eligibility criteria

Relevant articles had to be written in English and published in peer-reviewed journals. We included cross-sectional and longitudinal studies investigating joint biomechanics of unplanned movement tasks. Unplanned movement was defined as any time-constrained motor reaction (e.g., landing, stopping, or changing direction) performed in response to a stimulus presented during an ongoing baseline movement (e.g., walking, running, or jumping). Typical examples of unplanned movement tasks include COD into a previously unknown direction during walking, running, and sprinting, or single-leg landings with the landing leg being specified during the flight phase. Studies involving human participants of any age, sex, and athletic level were eligible, regardless of injury history or injury status at the time of testing. No restrictions were applied with respect to reactive task designs, stimulus modalities, or biomechanical outcome measures. Studies were excluded if they investigated (1) only pre-planned movement tasks (i.e., when the subsequent task or movement direction was known before baseline movement initiation or when sufficient time for advance planning was provided), (2) simple reaction tasks without a gross baseline movement, or (3) adaptations to mechanical perturbations (e.g., hinged drop-down flaps in walkways, hinged steps, or side-shearing floor panels).

After performing the search, all records were imported into Rayyan web application [29] and screened for duplicates. Duplicate records were identified using automated matching of title, author, and publication year, followed by manual verification, and removed prior to further screening. Subsequently, two authors (MS and AU) independently checked each article for inclusion with the following four steps: (1) title screening, (2) abstract screening, (3) full-text screening using the predefined exclusion criteria, and (4) final verification of eligibility. Disagreements between the two reviewers were resolved by a third author (JW), followed by discussion until consensus was reached.

In line with the scoping review framework, a formal risk-of-bias assessment was not performed. Scoping reviews aim to map the existing evidence and identify research gaps rather than critically appraise study quality, and therefore the inclusion of all relevant studies was prioritized.

### Data extraction and statistics

Two independent investigators (MS and AU) performed the data extraction using a standardised assessment sheet. We obtained the following data: sample size, participant characteristics (age, sex, anthropometry, health status, type of sport), baseline movement (movement pattern, approach speed or jump height), stimulus (type: e.g., arrow, sound, etc.; latency time, available response time), reaction task (movement pattern: e.g., COD, landing, jumping, stopping; specific characteristics such as the COD angle), assessed biomechanical outcomes (e.g., joint kinetics/kinematics, muscle activity, decision errors) as well as relevant cofactors (e.g., neurocognitive function). Furthermore, for each article, the year of publication, keywords, and the country of the first author were also recorded. All analyses were conducted in IBM SPSS Statistics (Version 27, 2021; Armonk, NY, USA) and R (Version 4.5.1, 2025; R Core Team).

### Evidence synthesis

Descriptive statistics for studies with kinematic, kinetic, and muscle activity data were applied, considering the moderating variables (e.g., unplanned movement tasks, stimulus, or participants). Data were presented in tabular form and analyzed qualitatively.

## Results

**Fig. 1:**
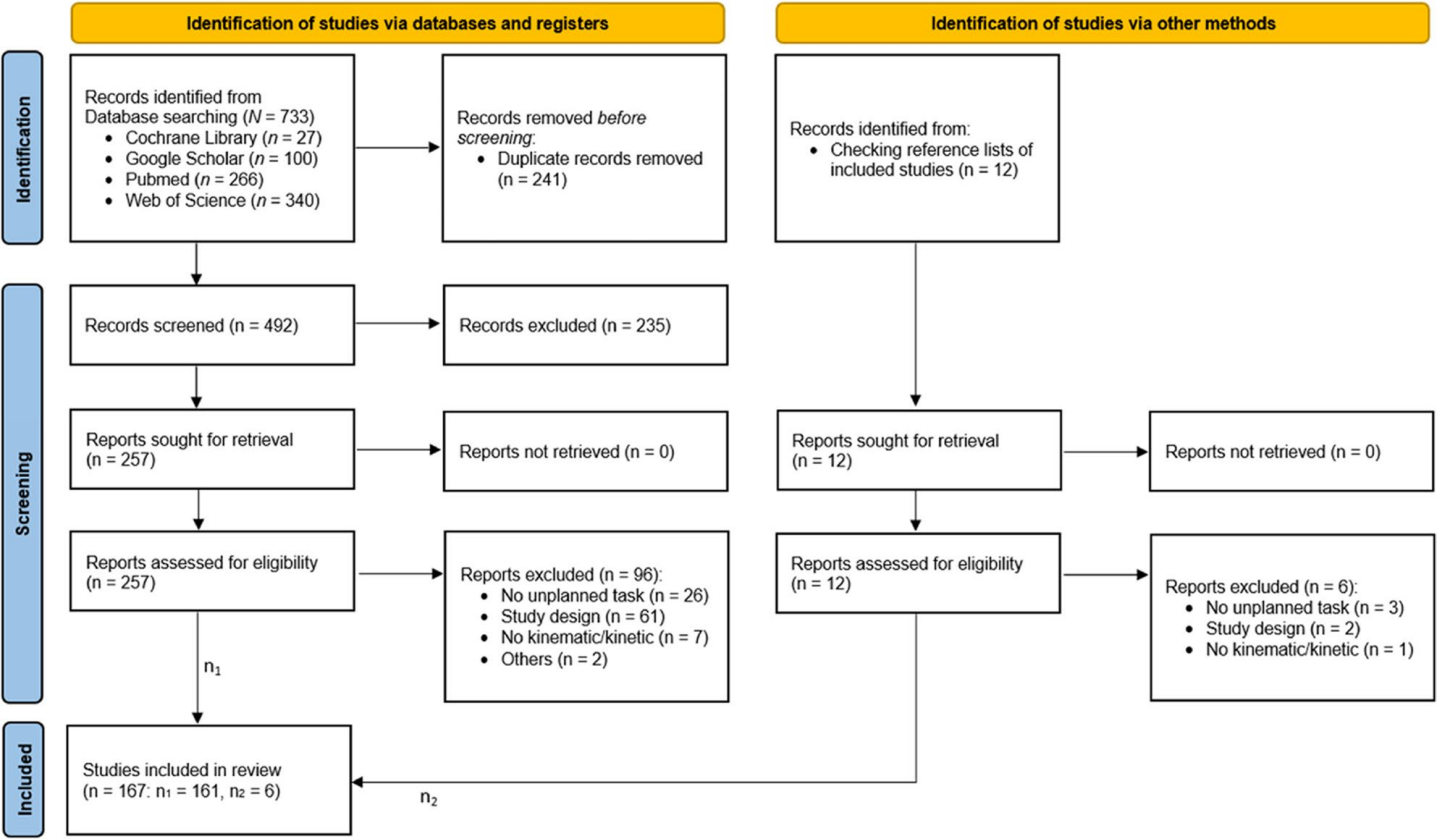
PRISMA flow diagram of the selection process. Source: Page MJ, et al. BMJ 2021;372:n71. 10.1136/bmj.n71. This workis licensed under CC BY 4.0. To view a copy of this license, visit https://creativecommons.org/licenses/by/4.0/

The literature search yielded a total of 733 studies (Fig. 1). After removal of duplicates and application of exclusion criteria, a total of 167 articles were deemed eligible. The study characteristics of the sample are presented in Suppl. Table 1.

### Publication year and country of origin of included studies

**Fig. 2:**
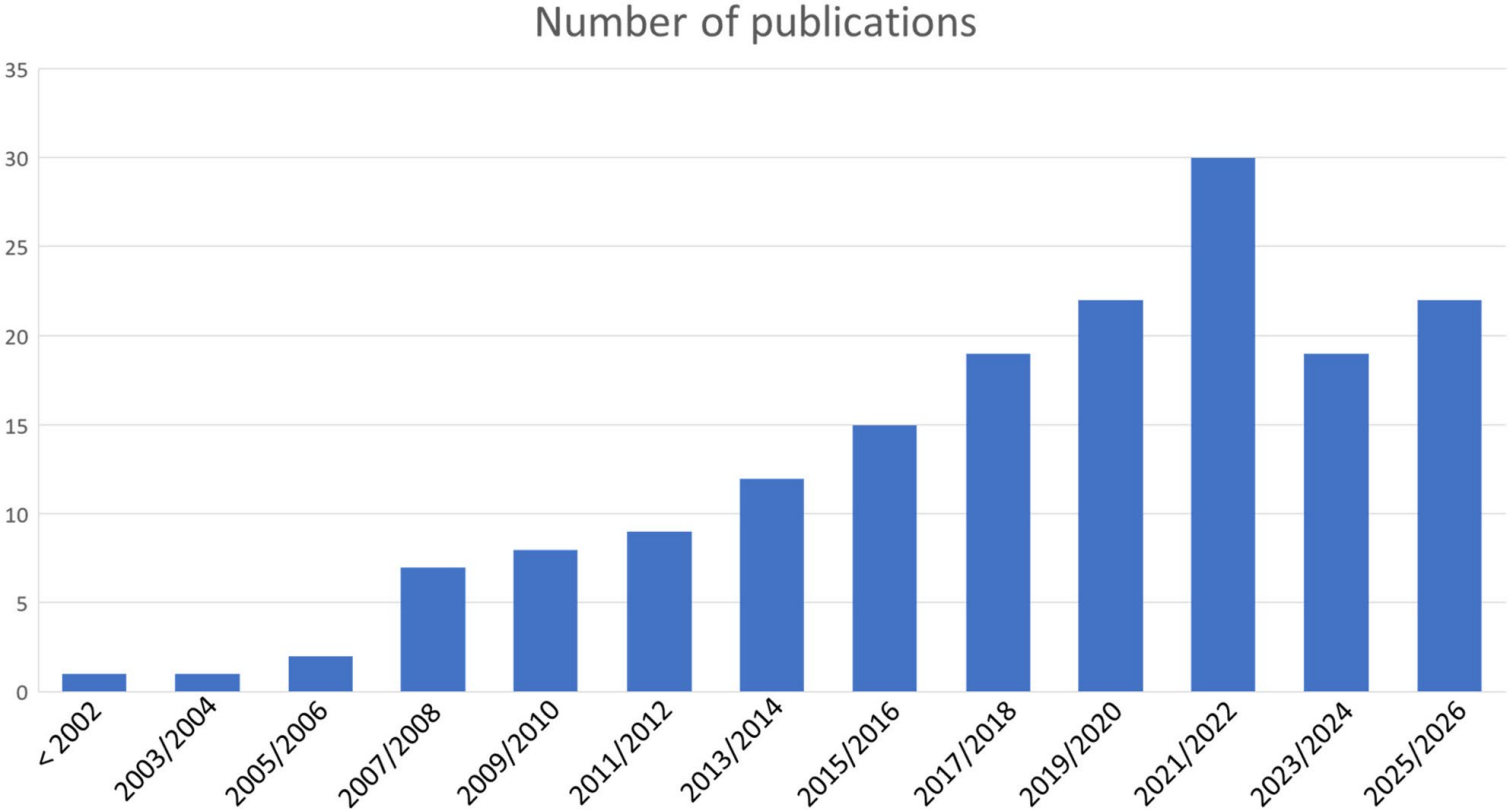
Temporal distribution of included studies. The figure presents the number of included studies per publication year

The number of studies involving unplanned movements increased over time. Only after the pandemic was there a decline in publications (Fig. 2).

**Fig. 3:**
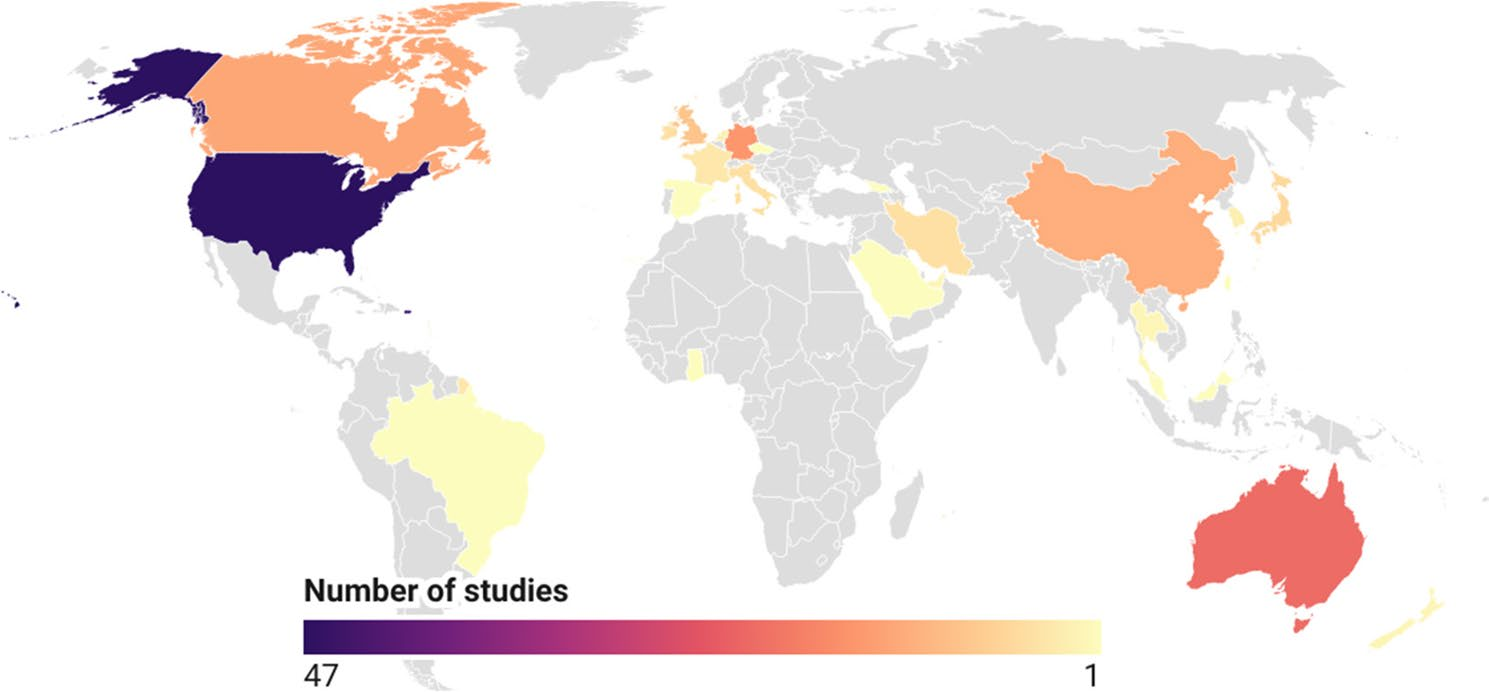
Global distribution of included studies. Countries are shaded according to the number of studies originating from each country. The map was generated using Datawrapper (Datawrapper GmbH, 2026, Berlin, Germany)

Most studies were conducted in the USA (47 studies), followed by Australia (21 studies), Germany (15 studies), Canada (13 studies), and China (12 studies). A total of 43 studies (26%) originated from European countries (Fig. 3), most frequently from Germany and the United Kingdom (9 studies).

### Standardized reporting framework

To improve comparability across studies, we structured the presentation of results according to four key domains that were consistently extracted from all included articles: (1) participant characteristics, (2) baseline movement, (3) stimulus properties, (4) unplanned movement task, and (5) biomechanical outcome measures. Below, each domain is summarized with standardized reporting of frequencies, ranges, and methodological commonalities across all 167 studies.

#### Participant characteristics

In total, 167 studies included 5,836 participants (57% women). Studies exclusively involving women (*n* = 58), men (*n* = 54), or both sexes (*n* = 55) were equally common. On average, the participants in the studies were 21.2 ± 3.6 years old (172.1 ± 7.4 cm, 68.0 ± 9.3 kg, BMI: 22.9). The study with the youngest participants had an average age of 9.6 ± 1.3 years. The oldest group of participants was 35.6 ± 12 years old. However, none of the studies involved participants older than 50 years.

**Fig. 4:**
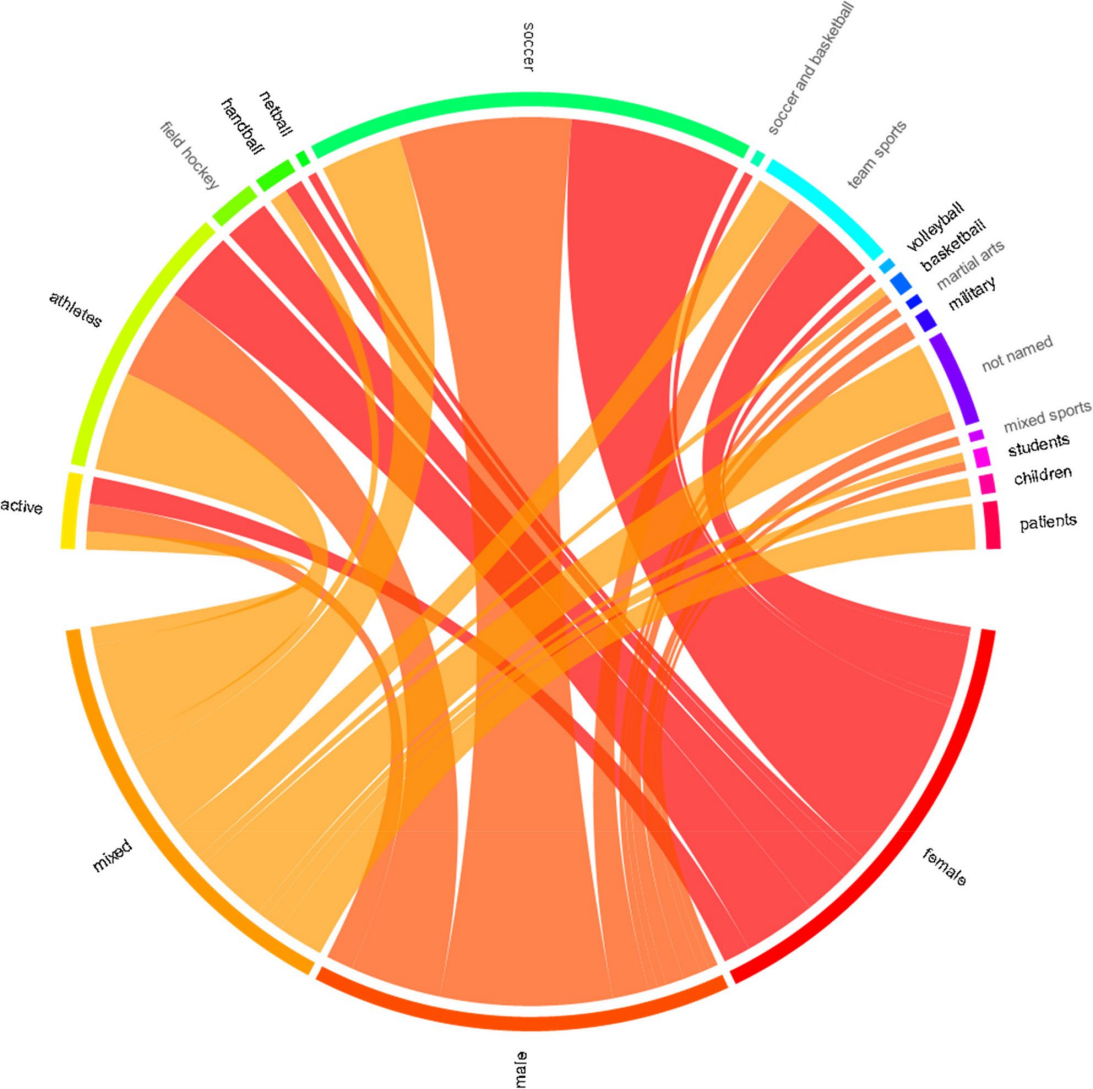
Chord plot of participant groups

The vast majority of the studies examined sports populations (80%, 133 studies). When stratifying by the type of sport (Fig. 4), soccer was, by far, examined most often (59 studies), followed by handball (7 studies), field hockey (6 studies), and basketball (4 studies). Several studies referred to participants simply as “athletes” without specifying the sport (34 studies) or included mixed “team sport” players (e.g., volleyball and basketball players; 18 studies). When grouped together, studies involving participants from one or more team sports accounted for 58.7% of all studies (93 studies). The remaining studies included patients (5 studies), students or children (4 studies) or military personnel (2 studies). In some studies, participant characteristics were either vaguely described (e.g., “active people”: 9 studies) or not reported at all (13 studies).

More than 80% of all studies involved healthy participants. Studies with non-healthy samples primarily investigated participants with anterior cruciate ligament rupture (ACL; 22 studies) or ankle sprain (4 studie); two studies each examined individuals with chronic ankle instability or concussion. One study analysed femoroacetabular impingement cases.

#### Baseline movement tasks

**Fig. 5:**
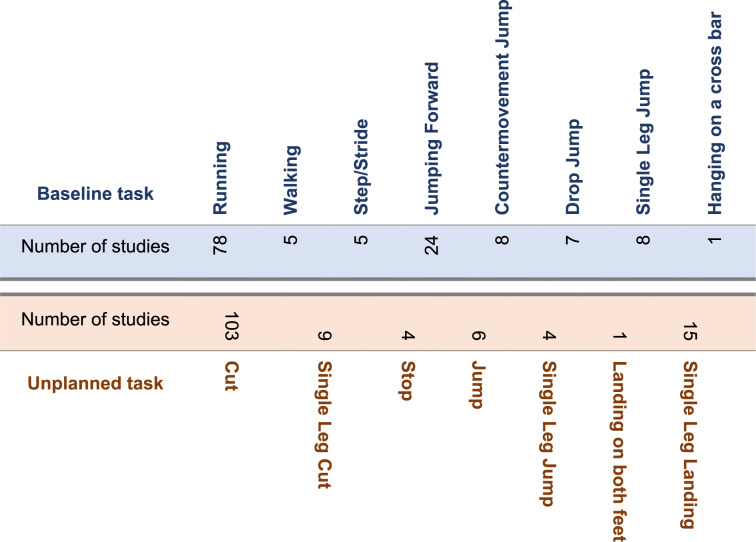
Baseline movement tasks and unplanned movement tasks (167 studies, individual studies examined several unplanned tasks)

The most frequently used baseline task (Fig. 5) was running (99 studies), followed by jumping forward (29 studies), countermovement jumps (10 studies), drop jumps (9 studies) and walking (6 studies). In studies using running as baseline movement, participants approached at a speed of 3.9 ± 0.8 m/s (14 ± 2.9 km/h). In jumping tasks, the mean distance of forward jumps was 100 ± 89 cm, the mean height in the vertical countermovement jump was 28.8 ± 2.2 cm, and the mean drop height in drop jumps was 32 ± 6 cm.

#### Cue/stimulus

Almost all studies used visual stimuli to elicit the unplanned movement component (99%, 165 studies). The most frequent types included flashing lights (84 studies), followed by symbols such as arrows or footprints (54 studies), videos (4 studies), real objects or people (11 studies), text (2 studies), and auditory signals (2 studies). Two studies additionally employed virtual reality. In most cases, the signal indicated the direction of the COD movement (e.g., side-cut or cross-over cut) or on which leg to land. The stimulus was static (e.g., symbol on a screen or light signal) in 89% of the cases and dynamic (video, virtual reality, or moving opponent/ball) in 10% of the cases. Latency between the stimulus trigger and its visual presentation (e.g., on a screen) was reported in only 13 studies, with an average of 75 ± 47ms (range: 12–120ms).

#### Unplanned movement task

**Fig. 6:**
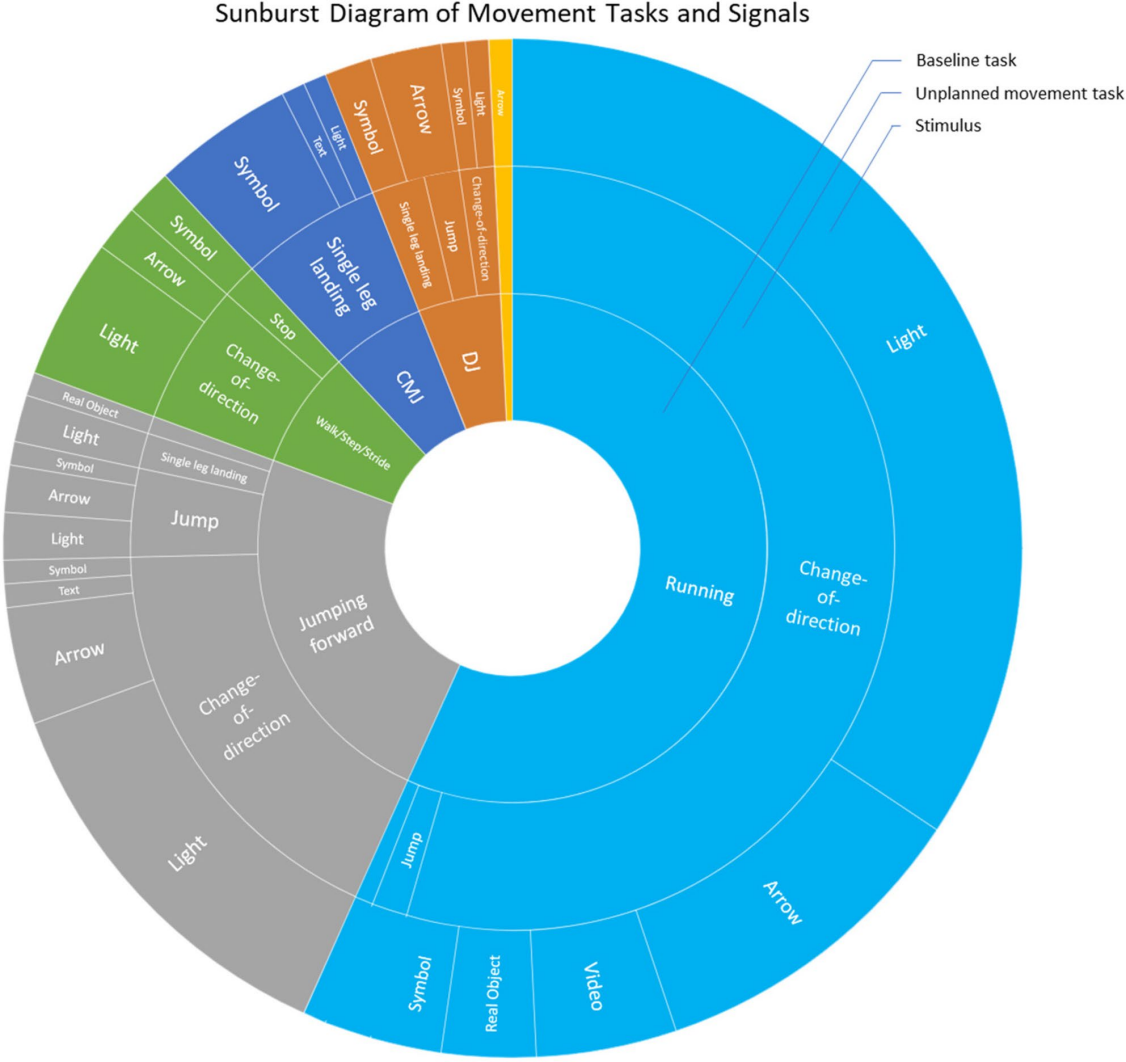
Sunburst diagram of movement tasks, unanticipated tasks and signals (The inner circle describes the baseline movement, the second circle shows which unplanned movement the participants should perform, and the outer circle indicates which signal was used in the studies to trigger the unanticipated movement)

In 77% of the studies (112 studies), the unplanned movement task consisted of a COD movement to the left or right. The COD angle was 45° in 96 studies and 90° in 17 studies. Deviating angles were reported in 21 articles: 20°to 40° in eleven studies, 55° to 75° in eight studies and 180° in two studies. Single-leg landings, e.g., following a drop jump from a box, were used in 19 studies (Fig. 6). Furthermore, in some cases, stops or one- and two-legged jumps were reported. The average response time available (stimulus presentation to required reaction) to the participants was 413 ± 143ms (100–760ms). In 100 studies, the participants had two movement options (e.g., land on left/right leg, COD to the left/right), 64 studies used three options (e.g., stop/side cut/cross-over cut), and 3 studies used four options (e.g., left/right/forward/vertical jumps).

#### Dependent variables

Across the included studies, a wide range of dependent variables was used to characterise unplanned movement tasks. These comprised four main categories: kinematic parameters (e.g., joint angles across lower‑limb joints), kinetic measures (ground reaction forces, joint moments), electromyographic data from lower‑limb muscles, and additional performance‑ or control‑related outcomes, such as centre‑of‑pressure measures, time to stabilisation, or decision‑making errors. This diversity reflects the multidimensional nature of reactive movement, encompassing mechanical loading, neuromuscular activation, postural control, and cognitive response demands.

### Commonly reported outcomes

Across the included studies, a consistent subset of kinematic and kinetic variables was reported most frequently. Kinematic measures were assessed in 144 of 167 studies (86%), with a predominant focus on the knee (140 studies; 84%), followed by the hip (94 studies; 56%) and ankle (64 studies; 38%). The most frequently analysed joint angles included knee flexion/extension and knee ab-/adduction, as well as hip flexion and ankle dorsiflexion/plantarflexion. These variables capture the core lower‑limb movement strategies during rapid deceleration and directional changes.

Kinetic variables were similarly common: ground reaction forces (GRF) were reported in 146 studies (87%), while joint moments were assessed in 93 studies (56%), most frequently at the knee. Key kinetic parameters included vertical GRF, loading rates, and knee extensor and abduction moments.

Clinically, these commonly analysed outcomes represent core markers of injury‑relevant loading patterns in unplanned movement tasks. Reduced hip and knee flexion (“stiff landings”), increased frontal‑plane knee motion, and elevated knee abduction moments are linked to ACL‑relevant loading. High GRF and rapid loading rates indicate increased mechanical stress on passive and active stabilising structures. Thus, the most frequently reported kinematic and kinetic variables reflect the biomechanical mechanisms most sensitive to risky movement adaptations under unanticipated conditions.

#### Kinematic outcomes

**Fig. 7:**
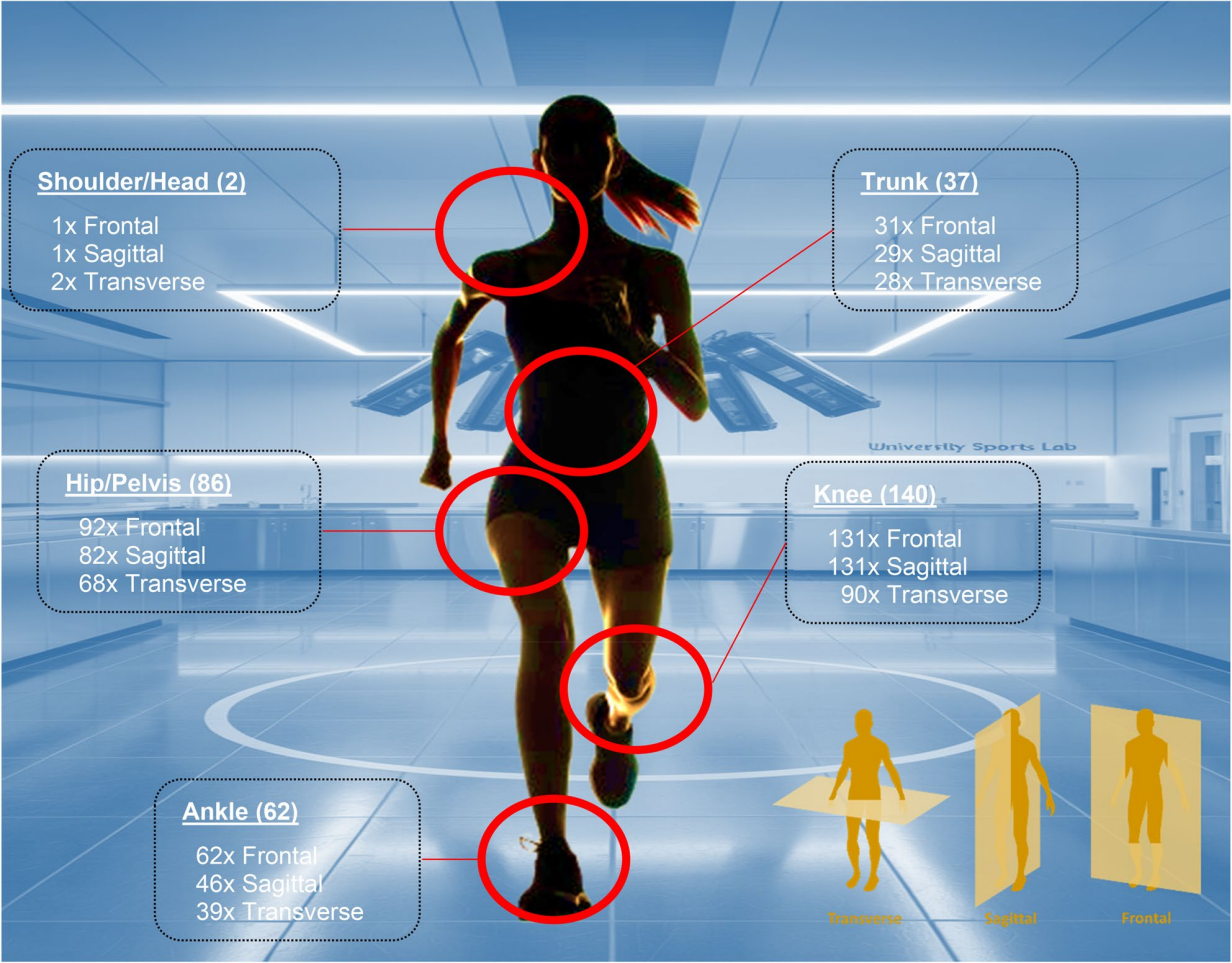
Joint and joint planes assessed in unplanned movement tasks (brackets show numbers of studies). Figure generated by AI (OpenAI DALL·E 3) and subsequently edited by the authors

Overall, 144 studies (86%) assessed joint angles (Fig. 7). Of these, 135 studies used 3D marker-based motion capturing, 5 studies applied 2D video recordings, and another 4 studies utilized both methods. Knee assessments focused predominantly on the frontal and sagittal planes (131 studies: adduction/abduction, extension/flexion). Also, sagittal plane movements—such as flexion and extension or dorsiflexion and plantarflexion—were most frequently examined in the hip (89 studies) and ankle (62 studies). Rotational movements in the transverse plane were the least investigated overall, appearing in only 76% of studies involving kinematic measurements.

#### Kinetic outcomes

Ground reaction forces, e.g., during COD or jump landings, were recorded in 146 studies (87%). For this purpose, 141 studies used force plates and five studies used pressure sensors. Joint moments were analysed in a total of 93 studies (93% knee, 67% hip, and 42% ankle). Other methods such as the measurement of time to stabilisation and centre of pressure path length were occasionally measured during single leg jump landings (22 of 167 studies). A minority of studies (*n* ≤ 3) considered energy absorption (W/kg), work (J/kg), peak pressure (KPa), loading rate (kg/s) or ankle stiffness (Nm/kg/°).

#### Electromyography and other methods

**Fig. 8:**
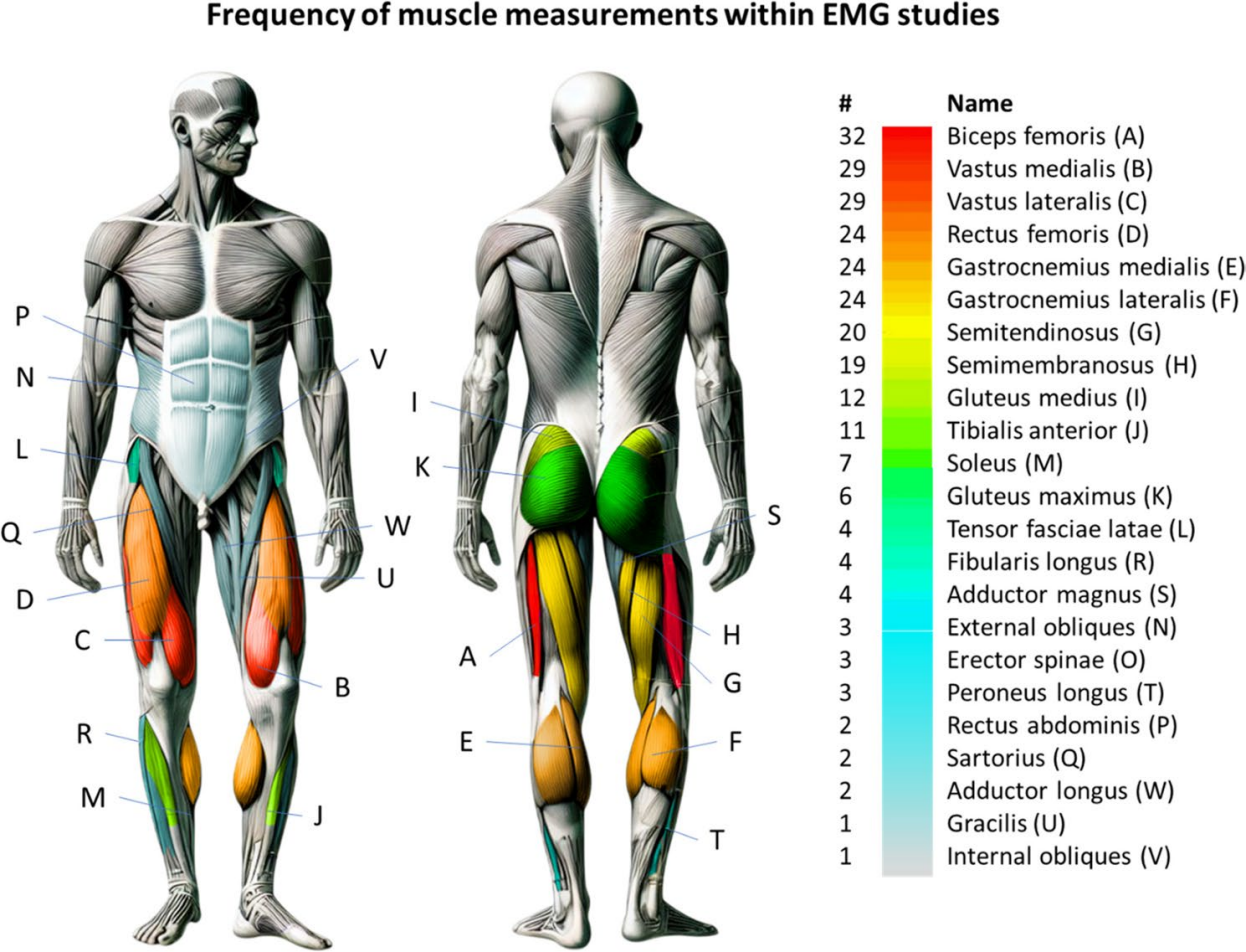
Muscles most frequently examined with EMG (total: *n* = 38 studies; six studies did not examine the biceps femoris and instead focused on other muscles.). Figure generated by AI (OpenAI DALL·E 3) and subsequently edited by the authors

Surface electromyography (EMG) was employed in 38 studies (23%). The biceps femoris was the most frequently measured muscle (32 studies), followed by the quadriceps femoris (29 studies; Fig. 8). The gastrocnemius was analyzed in 24 studies, while the other hamstring muscles were examined in 20 studies.

In addition to the typical kinematic and kinetic measurement methods using 3D video data and ground reaction forces, a small number of studies analysed other parameters and used alternative measurement instruments for this purpose. Eight articles described the application of inertial measurement units (6 studies), accelerometers or optical tracking systems. A few studies used magnetic resonance imaging (MRI) or electroencephalography (EEG). Furthermore, a very small proportion (6%, 11 studies) of the articles combined movement analysis with cognitive testing.

### Overall overview of included studies

**Fig. 9:**
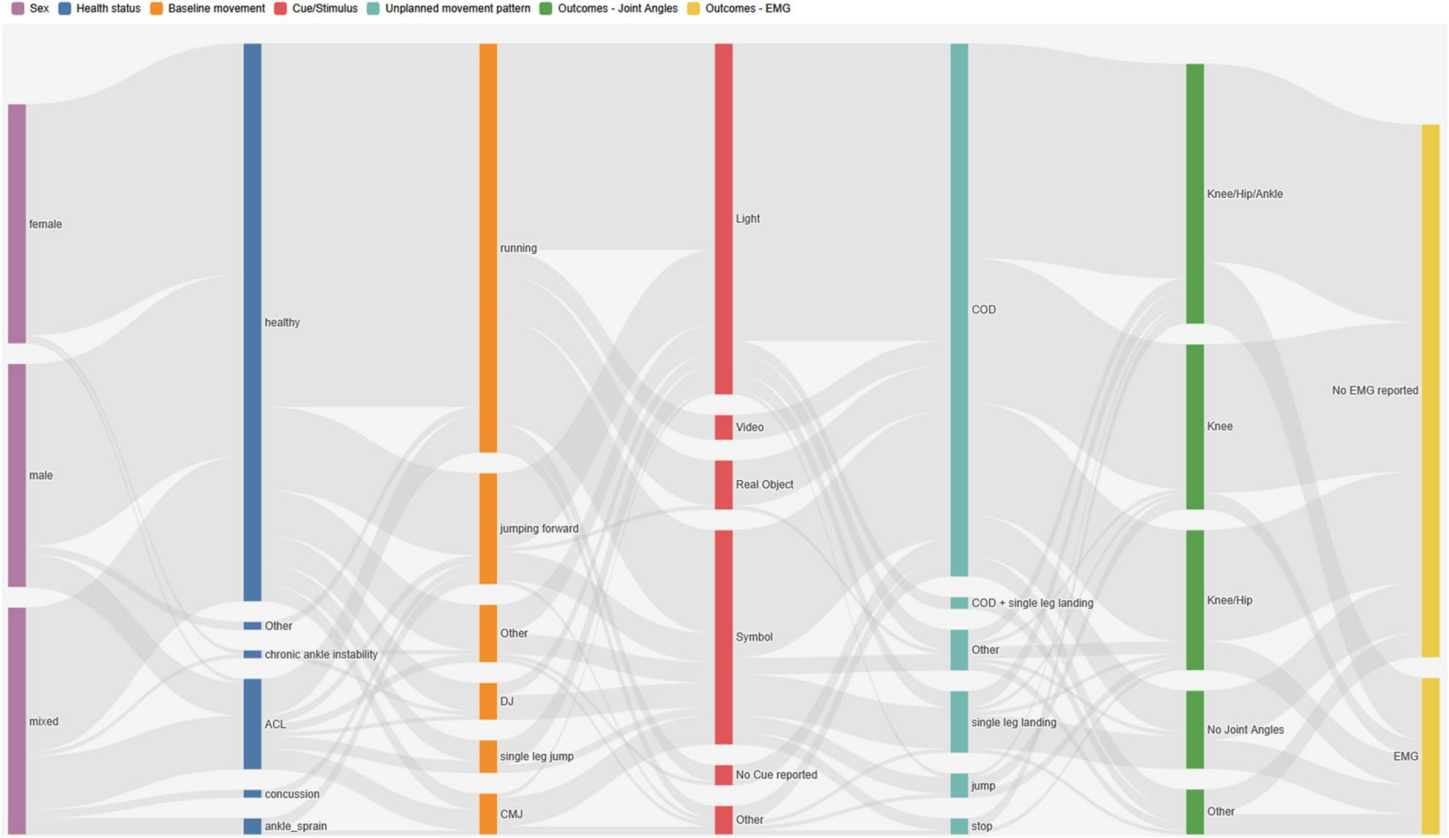
Sankey flow diagram illustrating the distribution of study characteristics across seven categorical variables. The interactive version of this figure (Supplementary File 2) allows users to select individual nodes and obtain a full breakdown of all associated categories across the remaining variables. To maintain a clear structure, studies reporting multiple outcomes were categorized according to the primary outcome described in the article or grouped as “other”

Overall, the included studies were predominantly conducted in healthy participants performing running-based COD tasks triggered by light signals, with joint angles representing the most frequently reported outcome measure (Fig. 9). Electromyography (EMG) was notably underrepresented. Studies focusing exclusively on female athletes with anterior cruciate ligament injuries were scarce in the literature.

### Keyword analysis

**Fig. 10:**
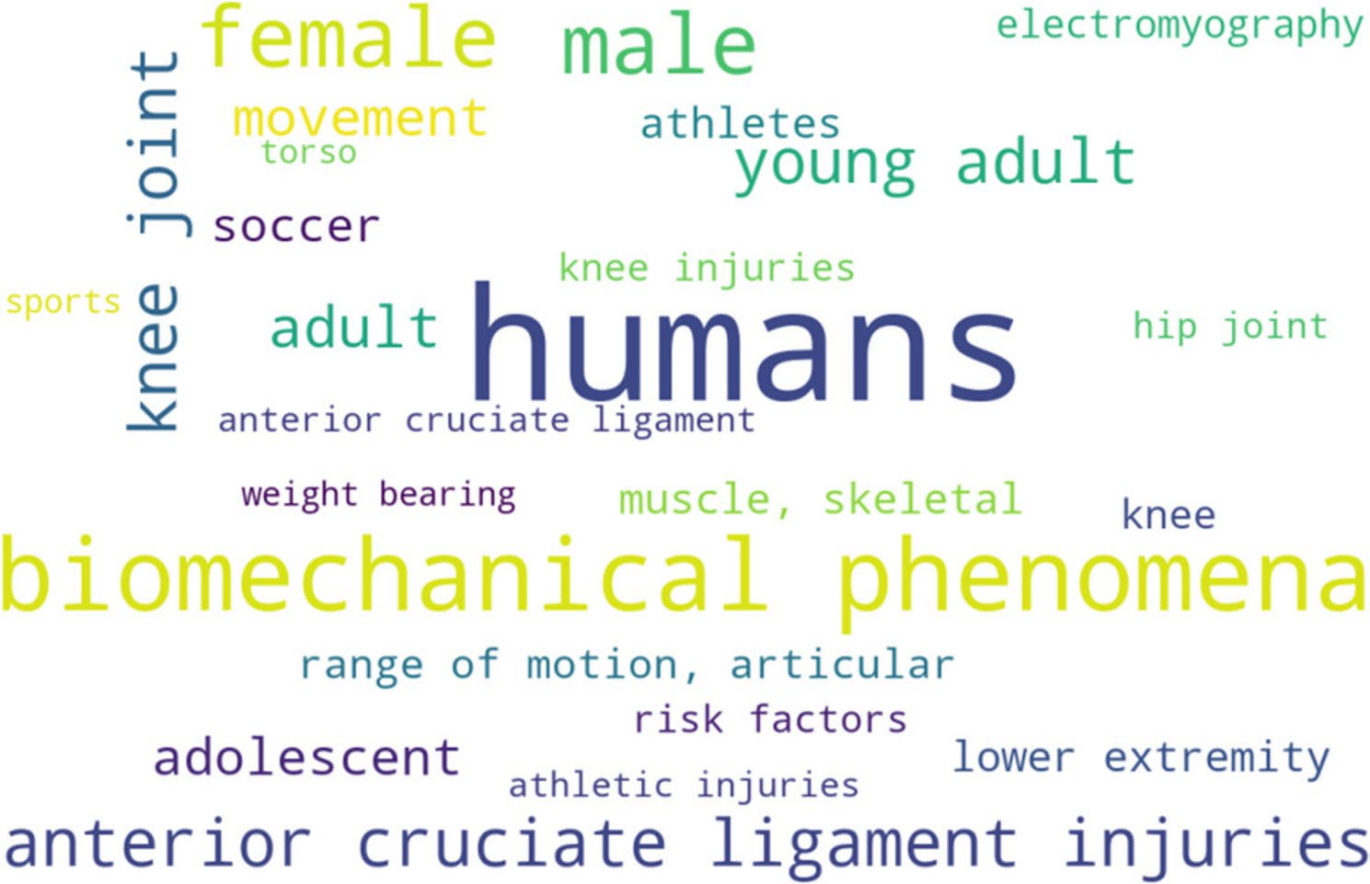
Word cloud showing the top 25 keywords extracted from CrossRef and PubMed MeSH across the selected studies

A keyword analysis based on author keywords and Medical Subject Headings (MeSH) identified 182 unique terms across the included studies. The 25 most frequent keywords were visualized using a word cloud (Fig. 10). The most common terms were humans (*n* = 135) and biomechanical phenomena (*n* = 119), followed by demographic descriptors such as female (*n* = 82) and male (*n* = 80). Several frequently occurring keywords referred to knee-related injuries and biomechanics, including anterior cruciate ligament injuries (*n* = 78), knee joint (*n* = 78), and knee injuries (*n* = 22). Additional common terms were related to movement characteristics and sport participation, such as movement (*n* = 50), soccer (*n* = 37), athletes (*n* = 29), and electromyography (*n* = 24). Overall, the keyword distribution indicates a strong focus on biomechanical aspects of lower-extremity movement and knee injuries in athletic populations.

## Discussion

The enrichment of biomechanical testing paradigms with neurocognitive components represents an intriguing topic with applications in both sports and daily living. Adding reactive decision-making to traditional assessments opens new avenues for studying athletic performance, injury risk, and functional capacity (e.g., in older adults). The growing consensus on the relevance of tasks capturing unplanned decision-making is impressively underlined by the results of our scoping review: we identified a total of 167 studies investigating reactive movement tasks. Despite the high quantity of articles, our main findings are (a) substantial methodological heterogeneity in both study design and outcome measurement, and (b) potentially limited transferability to real-world settings.

The relevance of studies investigating unplanned movements has increased substantially over the past decades. Whereas in 2002 hardly any studies on this topic could be found, around 2025 approximately 20 studies were being published each year. Most of these studies originate from the United States and Europe. In contrast, comparatively few publications come from China, the world’s second-largest economy, which ranks only fifth worldwide in terms of the number of publications in this field. Overall, the topic is represented globally in the scientific literature—in total, 25 countries (based on first authorship) have conducted studies on unplanned movements. Both the global increase in the number of studies and the wide range of contributing nations suggest a growing clinical interest in this field.

### Movement patterns

The most often investigated movements of the sampled studies were COD (82%, 137 articles). This is plausible as they count among the commonest actions in sports. Bloomfield et al. [30] reported a total of 720 turns per soccer match. A notably high number of COD has also been shown for handball, with around 60 high-speed turns (> 2.5 m/s) per game [31]. Interestingly, COD movements only account for a small percentage of injuries in handball (8%; [32]). Basketball, however, presents a different picture: Achenbach et al. [33] demonstrated most non-contact injuries to occur during feinting, especially during COD and sprints. Tosarelli et al. [34] made similar observations: Using video analyses in men’s basketball, they found almost half of all ACL injuries during COD movements. Also, in soccer, the most frequently represented sport in our study (35%), COD, landings, and combinations of both movements are the typical movement patterns associated with non-contact injuries [35, 36]. In this context, Klein et al. [37] reported that 17.9% of knee injuries and 15.1% of ankle injuries could be attributed to these movement patterns.

In 96 of the 167 COD studies, a COD angle of 45° was used. This is of importance because in real sports situations, turning angles may be significantly different. Dos Santos et al. [38] applied automatic tracking to determine the COD angle of 22 soccer players, classifying 63–70% of the turns as high-angled (120–180°). With the same method, Deutsch et al. [39] analysed athletes competing in the top German soccer league. They found more than 65% of all CODs in a range between 120 and 180° while angles of 45° occurred in only 6% of all cases. Conversely, Bloomfield et al. [30] and Morgan et al. [40] argued that most COD (approx. 80%) occurred at less than 90°. However, they did not specify the exact mean angle of the examined COD. In sum, the COD angle of 45°, used in a large portion of the current literature, may underestimate the actual demands of real-game situations [41].

Theoretically, higher angles could cause larger knee joint moments as they require a different foot strike and a greater deceleration shortly before the COD [42]. Clinical studies by Boden et al. [23] showed that spontaneous decelerations are particularly responsible for non-contact ACL injuries. This fits with data of Dos Santos et al. [43] showing the greatest risk of injury at a COD angle of 90°, which was related to a ‘angle-velocity trade-off’. Also, Li and Qian [44] demonstrated a progressive elevation of peak knee abduction moment and dynamic valgus at increased COD angles, with a critical nonlinear threshold at 90°, corresponding to a substantially elevated ACL injury risk. Future studies should therefore consider investigating higher COD angles.

A similar experiment-practice discrepancy may exist regarding approach speed. The average running velocity of the 99 running studies using run-and-COD tasks was 3.9 m/s. COD in soccer usually occur at run-up speeds of less than 5.5 m/s [38, 45]. Nevertheless, studies by Deutsch et al. [39] also show that 10% of the COD movements have a run-up speed of over 7.7 m/s. While higher running speeds may increase quadriceps loading and, consequently, injury risk [46], it is still unclear at which COD velocity the most frequent load injuries occur. Notwithstanding, it seems advisable to include both moderate and high approach speeds in future studies to reflect real sports demands.

The timing of the stimulus is the key variable defining whether a task is executed under anticipated or unanticipated conditions. Reported reaction times in unplanned tasks ranged around 413 ± 143ms (100–760ms), indicating substantial between-study variability. While approach speed determines the distance available to respond, it does not inherently change the reaction time; in other words, reactive COD tasks can be designed with consistent time-to-react intervals regardless of approach speed. To allow meaningful comparison across studies, it is essential that the methodology reports the available reaction time (including any device‑related latency). Moreover, in addition to movement speed, researchers should specify the exact distance between timing gates and the point of ground contact, which does not necessarily correspond to the beginning of a force plate.

Conversely to the high representation of unplanned COD in the available literature, jump landings were less frequently examined. The overall portion looking at baseline jump tasks was about 34% (56 articles) of all studies, which seems satisfactory at first glance. However, while ACL injuries in team sports are most frequently associated with COD movements (26–70%), landing situations account for a substantially larger proportion of ACL injuries in overhead sports such as volleyball and badminton—where 57–82% of injuries occur during landings [47]. Consequently, landing tasks are of high scientific relevance for these sports. Nevertheless, landings also represent a relevant injury mechanism in team sports: in basketball, 22% of ACL ruptures and 38% of all injuries occur during failed landings [33, 34]. In handball, around 30% of all injury-related movements were attributed to landings [32]. However, two thirds of all found landing studies added a subsequent COD movement (35 articles) or a further jumping task (9 articles), thus placing the focus on the follow-up movement instead of the landing safety. The small number of studies examining unplanned single- or double-leg landings (12%, 20 articles) in isolation is surprising, as jumps and landings are ubiquitous in many sports, being associated with a high risk of injury. Accordingly, a clear gap exists in the literature concerning the role of unplanned decision-making during landing tasks. Therefore, future studies should increasingly investigate jump landing tasks, particularly in overhead sports (9%, 16 studies), which have been little researched to date.

Finally, very few studies investigated stop tasks (3%, 5 studies). This seems consistent with actual reality. Klein et al. [37], for example, report that most injuries to knee and ankle occur during landings and COD. Achenbach et al. [33] also state that only 6% of all basketball injuries were caused by stops. However, in other populations—such as clinical groups or older adults—stop tasks may be of considerably greater relevance.

### Visual cue

Most studies (42%, 84 articles) used simple light cues to indicate the unplanned COD direction or the landing leg, whereas 32% (54 articles) used symbols (e.g., foot sign or arrow). Only 9.6% of all articles (16 articles) used videos or real human stimuli as a trigger. Two of the included studies employed virtual reality–based cues. Interestingly, previous research suggests that human reaction times can vary depending on the type of visual cue. Symbols, for example, can be recognized more quickly and easily than words or letters [48]. Even the color of the signal can affect reaction time as faster reactions were observed for red than for green or blue signals [49]. In addition, changes in light and colour could be processed more quickly than body movements [50]. However, athletes may be more accustomed to interpreting sport-specific body language and less familiar with abstract visual cues. It is therefore questionable whether symbols and light signals can adequately reflect situations from team sports [51]. In sum, future studies should be geared to further examine the influence of signalling on reaction time and the kinematics and kinetics of movements. In addition, more realistic cues, e.g., real-life video scenes or animations may be used. In the future, technologies such as augmented and virtual reality may provide ecologically valid settings for the assessment of unplanned movement tasks.

### Examined joints and biomechanical outcomes

Our analysis showed that the knee was the focus of most studies (83%, 139 studies), while the hip (56%, 94 studies) and ankle (41%, 68 studies) were investigated less often. The prioritization of the knee was to be expected due to its pivotal biomechanical significance and the considerable loads it absorbs during rapid athletic movement. The knee joint plays a central role in the kinematics of the lower limb and is significantly involved in force transmission and stabilization between hip and ankle, making it a key factor in neuromuscular control during sudden changes in movement [18, 52, 53]. An excessive valgus position of the knee in the frontal plane can lead to increased stress on the passive structures [54] and has been discussed as a potential risk factor for ACL injury [55]. However, recent evidence may suggest that knee valgus and knee internal rotation are unlikely to be primary contributors to ACL injury, but may rather represent changes in post-injury kinematics [56, 57]. Instead, non-contact ACL injuries are frequently associated with a stiff landing strategy, characterized by low hip and knee flexion angles, and increased knee extensor moments [58, 59]. Accordingly, studies investigating ACL injury mechanisms may benefit from focusing particularly on sagittal and frontal plane kinematics and kinetics of both the knee and hip joints [60]. From a clinical perspective, these findings suggest that assessment and monitoring of landing mechanics during unplanned movement, e.g., hip and knee joint function, could be relevant for injury prevention and rehabilitation strategies, though further research is needed to translate these biomechanical insights into targeted interventions.

Despite the focus on the knee joint, we found many studies investigating proximal (hip) and distal (ankle) joints. The hip is central to lower limb stabilization, particularly during movements requiring rapid directional changes and complex kinematics, and its function significantly influences knee joint movement [61, 62]. Restrictions in hip rotation have been associated with an increased risk of injury in neighboring joints, which is particularly relevant during lateral movements and stop jumps [63]. In addition, compensation through the proximal joint chain, such as after an ankle injury, could elevate mechanical load on the knee and hip, thereby increasing the risk of injury [64–66].

Regarding kinetics, many studies (87%, 146 studies) used vertical ground reaction forces and peak pressures as indicators of the impact and mechanical loading, while joint moments were considered in a smaller number of studies (59%, 98 studies). Nevertheless, the evaluation of the resulting loads on joints and muscles may provide valuable insights for the development of preventive measures. Only a few studies (23%, 38 studies) on unplanned movement measured skeletal muscle activity using EMG. Within EMG studies, the most frequently examined muscles were biceps femoris (84%, 32 studies), vastus lateralis/medialis (76%, 29 studies), and rectus femoris (63%, 24 studies). This observation is of central importance as the hamstrings and quadriceps play a key role in controlling knee extension/flexion and preventing excessive knee valgus [57, 67]. The quadriceps and gastrocnemius can increase ACL loading by generating anterior tibial shear forces, particularly near full knee extension, whereas the hamstrings and soleus help unload the ACL via posterior shear [68]. Also, the gluteus medius consistently counteracts knee valgus moments, providing greater ACL protection than any other muscle [69]. A systematic review of Georgoulis et al. [70] showed athletes exhibit decreased quadriceps and/or increased hamstrings EMG activity after ACL reconstruction despite return-to-sport. Increased hamstrings activation could thus represent an adaptive response to stabilize the knee joint by counteracting excessive anterior tibial translation and valgus stress, with potential implications for ACL injury prevention and safe return-to-sport paradigms [71]. For these reasons, upcoming studies should consider adding EMG measurements when assessing unplanned movement tasks in the context of rehabilitation and injury prevention.

### Additional diagnostic approaches

Very few studies have applied additional diagnostic measures such as MRI (Magnetic Resonance Imaging) or EEG (Electroencephalography). Most frequently, cognitive tests, for example the Stroop test, were used as additional predictors to explore potential biomechanical associations (8%, 12 studies). Several reviews [10, 17–21, 72] indicate that cognitive demands and function are directly related to biomechanical indicators associated with ACL injury risk. Lower cognitive performance tends to be linked to movement patterns that increase injury risk during cognitively demanding tasks [73]. Accordingly, it appears valuable to incorporate these factors in future research. Potential approaches have already been demonstrated in individual studies using complex stimuli [74], dual-task paradigms [75], return-to-sport testing [76, 77] or mental fatigue protocols [78].

### Studied populations

Of the studies reviewed, 54 focused exclusively on males, 58 examined only females, and an additional 55 investigated mixed-sex groups. Overall, female participants accounted for 57% (n_♀_ = 3,322) of all participants (overall 5,836 participants). This seems adequate as overall, women show a greater risk of joint injuries than men due to anatomical, hormonal and neuromuscular differences [79, 80]. Women experience distinct biomechanical loading characteristics due to their physique, including a wider pelvis and altered joint angles, especially during movements that require rapid COD [81, 82]. These structural differences influence joint mechanics and increase the risk of injury, especially in sports or activities that place high demands on movement coordination and stability. The number of ACL ruptures is three times higher in women than in men [83]. In addition, the number of injuries in women has increased significantly over the last 20 years [84]. Unplanned decision-making tests may, therefore, be particularly suitable in women.

A large proportion of studies (67%, 112 studies) investigated young, healthy individuals with an average age between 17 and 25 years. On the one hand, the focus on healthy young adults can be explained by the high level of physical performance and the low prevalence of chronic diseases or muscular dysfunctions, which simplifies the analysis of biomechanical mechanisms. On the other hand, the incidence of lower limb injuries, particularly knee injuries, is relatively high in this age group due to frequent sporting activities and the associated mechanical stresses [85, 86]. Studies by Mainar et al. [84] and Sanders et al. [87] show that the greatest risk of knee injuries is between the ages of 15 and 25. Accordingly, the investigations in most studies on this target group appear to be justified. However, the current focus could limit the transferability of the results to other population groups or specific clinical contexts. In particular, the prioritization of young, physically active individuals raises the question of the extent to which these findings can be applied to older populations. Age-related changes in neuromuscular control, such as reduced intersegmental coordination (e.g., foot-shank coordination; [88]), sarcopenia [89, 90], and the increased loss of type II muscle fibers [91], increase the risk of injury and reduce functional independence [36, 92]. The risk of injury and the consequences of those are far higher in older groups than in young, trained recreational athletes. The findings from unplanned movement tasks, in particular, could provide decisive information for recognizing injury and fall risks in older people. Such knowledge could also have a direct positive influence on the functional independence, fear of injury and sovereignty in everyday life. With regard to the increased risk and high potential importance for older adults, the transferability of the results of our scoping review remains unclear—especially since not a single study has investigated this population group. Currently, it remains challenging to derive specific guidance for research or for implementing screening, preventive, or rehabilitative strategies for older adults and patient populations in the context of unplanned movements. Thus, further studies on groups of older people are urgently needed.

### Keyword analysis

The predominance of keywords related to knee injuries, particularly anterior cruciate ligament (ACL) injuries, and biomechanical phenomena highlights the clinical importance of understanding lower-extremity mechanics during unplanned movements in athletic populations. This focus underscores the need for targeted strategies in injury prevention, rehabilitation, and performance optimization that account for the sudden and unpredictable nature of sports tasks. The frequent occurrence of demographic terms such as “female” and “male” further suggests that sex-specific risk factors and interventions are relevant. Overall, the keyword profile demonstrates strong translational relevance, showing how biomechanical insights from studies of unplanned movements can inform evidence-based approaches to reduce the incidence and severity of sports-related knee injuries.

### Clinical implications

This scoping review highlights a heterogeneous body of literature on unplanned movement tasks. Most studies combine 3D motion analysis systems (e.g., marker-based motion capture) with force diagnostics (e.g., force plates). Knee and hip angles, as well as joint moments in the frontal and sagittal planes, are particularly informative for assessing injury-relevant risk factors, e.g., with reference to ACL injuries. Muscle activity of key stabilizing muscles—quadriceps, gastrocnemius, gluteus medius, hamstrings, and soleus—is commonly analyzed to evaluate movement control and knee stability.

Depending on the population and sport, either landing tasks (e.g., in overhead sports) or COD tasks (e.g., in team sports) are recommended as unplanned tasks, due to their high frequency and relevance for injury risk. COD tasks are typically preceded by running as a baseline movement and they should involve high speeds and large COD angles (e.g., up to 90°). For unplanned tasks, reaction times of approximately 400ms between stimulus and movement initiation are advisable in order to trigger decision-making under time pressure.

Future studies should incorporate more ecologically valid stimuli, such as real opponents, videos, or virtual reality environments. Additionally, cognitive load may provide valuable insights into potential injury risks. Although females aged 15–25 are at elevated risk for ACL injuries, other populations, such as older adults, should also be investigated to address potential research gaps. Furthermore, future research should move beyond the predominant focus on running-based COD tasks and consider a broader range of movement patterns, such as single-leg landing or jumping tasks. Simultaneous assessment of joint angles and EMG is strongly encouraged, as the combined neuromechanical perspective remains underrepresented in the current literature. In particular, a study examining female athletes with ACL injury performing single-leg landing tasks in response to video- or real object-based cues, while simultaneously capturing joint angles and EMG, would directly address several of the identified gaps and represents a highly warranted next step for the field.

## Conclusions

The investigation of unanticipated movement is a trending topic in biomechanical research. While many studies address relevant movement patterns and participant groups, they are often conducted under artificial laboratory conditions with simplified tasks and stimuli. Only a few available trials incorporated more ecologically valid approaches, such as realistic video cues, virtual reality, or interactions with real opponents. This raises concerns about the generalizability of the findings. Furthermore, the role of cognitive factors in test outcomes and injury risk remains unclear, as does the transferability of results to other populations, such as older adults. Future research on unplanned movements may benefit from a stronger focus on older populations, larger cutting angles, and the use of more realistic stimuli, such as virtual or augmented reality, to enhance ecological validity and clinical relevance.

## Search strategies for each database

### MEDLINE (via PubMed)

- (unplanned OR unanticipated) AND (“change of direction” OR cut OR cutting OR land* OR jump* OR stop* OR step*) AND (EMG OR electromyography OR “muscle activity” OR kinetics OR kinematics)

### Web of Science (Core Collection)

- ((((TS = unplanned) OR (TS = unanticipated)) AND ((((((TS = “change of direction”) OR (TS = cut*)) OR (TS = land*)) OR (TS = jump*)) OR (TS = stop*)) OR (TS = step*))) AND (((((TS = EMG) OR (TS = electromyography)) OR (TS = “muscle activity”)) OR (TS = kinetics)) OR (TS = kinematics)))

### Google Scholar

- (unplanned OR unanticipated) AND (“change of direction” OR cut* OR land* OR jump* OR stop* OR step*) AND (EMG OR electromyography OR “muscle activity” OR kinetics OR kinematics)

### Cochrane Library

- (unplanned OR unanticipated) AND (“change of direction” OR cut OR cut* OR land OR land* OR jump OR jump* OR stop OR stop* OR step OR step*) AND (EMG OR electromyography OR “muscle activity” OR kinetics OR kinematics)

## Supplementary Information

Supplementary Material 1.

Supplementary Material 2.

## Data Availability

The datasets used and/or analysed during the current study are available from the corresponding author on reasonable request and are available in the following databases: Web of Science, MEDLINE (PubMed), and Cochrane Library.

## Abbreviations

ACL: Anterior cruciate ligament
AI: Artificial intelligence
COD: Change of direction
EEG: electroencephalography
EMG: Electromyography
MRI: Magnetic Resonance Imaging
