# Supplementary material for: Unplanned movement tasks requiring rapid decision-making: a scoping review of study characteristics and methodological approaches
Source: BMC Sports Sci Med Rehabil. 2026 May 8;18:262. doi: 10.1186/s13102-026-01682-0 (PMC13231710; doi:10.1186/s13102-026-01682-0)
Supplement: Supplementary file 2 — Supplementary Material 2. [file 13102_2026_1682_MOESM2_ESM.html]

Sankey – Study Characteristics


# Study Characteristics – Sankey Flow

Sex → Health status → Baseline movement → Cue/Stimulus → Unplanned movement pattern → Outcomes (Joint Angles) → Outcomes (EMG)  |  Klick auf einen Knoten = vollständige Aufschlüsselung

## Aufschlüsselung

Klicke auf einen Knoten im Sankey-Plot

← Knoten anklicken
